# Characterization of antigens of *Enterobius vermicularis* (pinworm) eggs

**DOI:** 10.1038/s41598-022-18303-8

**Published:** 2022-08-24

**Authors:** Y. B. N. Sanduni De Kostha, Sisira L. Pathirana, Shiroma M. Handunnetti, Sharmini Gunawardena

**Affiliations:** 1 Institute of Biochemistry, Molecular Biology and Biotechnology, University of Colombo, Colombo, Sri Lanka; 2 Department of Parasitology, Faculty of Medicine, University of Colombo, 25, Kynsey Road, Colombo 08, Sri Lanka

**Keywords:** Isolation, separation and purification, Microscopy, Immunological techniques, Antibody generation, Antibody isolation and purification, Immunoblotting, Immunology

## Abstract

Enterobiasis (pinworm infection) caused by *Enterobius vermicularis* is a common parasitic infection prevalent worldwide especially in children. Infection is diagnosed by microscopic detection of *E. vermicularis* eggs on perianal swabs. This study aimed to characterize the antigens of *E. vermicularis* eggs as a preliminary step towards identifying diagnostic targets for detection in infected individuals. The study was conducted between October 2019 and February 2020, following approval from Ethics Review Committee of the Faculty of Medicine, University of Colombo (EC-19-034). *E. vermicularis* eggs were harvested from perianal swabs using acetone and purified with 1× PBS (pH 7.2). A portion of eggs was used for preparing antigen slides, while the rest were sonicated and vortexed with glass beads and inoculated subcutaneously (with weekly booster doses) into a Wistar rat for developing antibodies. Blood drawing from rat was done weekly for 5 weeks. Confirmation of the presence of antibodies was done by surface immunofluorescence against eggs on the antigen slides. Protein bands were determined using SDS-PAGE assay and immunogenic antigen bands were determined by reacting with antiserum after immunoblotting. The band sizes of the proteins were determined against corresponding bands of a protein ladder. Surface immunofluorescence was positive with serum obtained from day 14 post-inoculation from the Wistar rat as well as that obtained from a person with chronic enterobiasis. The most prominent and immunogenic protein bands identified from egg antigens were 21 kDa, 66 kDa, 83 kDa, 96 kDa, 112 kDa, 121 kDa, 140 kDa and 151 kDa. Methods used in this study were effective in obtaining *E. vermicularis* egg antigens which were immunogenic. Furthermore, surface antigens of intact eggs reacted with antibodies developed against crushed egg antigens. These findings may pave the way for the development of effective immunodiagnostics.

## Introduction

*Enterobius vermicularis* (pinworm) is the causative nematode parasite of enterobiasis (pinworm infection) in humans. Enterobiasis is prevalent worldwide occurring in both temperate and tropical regions with over 1 billion people estimated to be infected^1,2^. Prevalence of infection is high among young children, and it is commonly a family or group infection, particularly in overcrowded living conditions with poor personal hygiene. An island wide survey conducted recently in Sri Lanka revealed the prevalence of enterobiasis to be 22% among schoolchildren attending Grades 1–2^3^. Infected persons may remain asymptomatic or develop pruritus of the perianal region (and/or vulvovaginitis in females) leading to insomnia, restlessness, irritability, enuresis, and secondary infection of scratched skin^2,4–6^. And occasionally, it may give rise to abdominal pain, vomiting and appendicitis^7^. Although effective medications are available, control of enterobiasis is difficult because of autoinfection, reinfection, incomplete cure of infected people, and its high transmissibility^2^.

*Enterobius vermicularis* has a simple life cycle^4^. Adult worms live in the proximal colon of infected patients and the gravid females migrate to the perianal region, especially at night, to deposit their eggs on the perianal skin. Local irritation caused by the migration of worms and their sticky eggs will provoke intense pruritus. Scratching of the perianal region will facilitate transfer of eggs on to the fingers as well as help in dispersing eggs into the environment. These eggs become infective within 6 h of oviposition and are transmitted via ingestion (or by inhalation of airborne eggs which are subsequently ingested) either directly from contaminated hands or through fomites, such as bedding and the utensils used by an infected individual^4^.

Diagnosis of enterobiasis is done routinely by microscopic detection of the characteristic eggs of *E. vermicularis* in adhesive perianal swabs. Occurrence of egg deposition at night entails the perianal swabs to be obtained early morning prior to washing of the perianal region. Furthermore, deposition of eggs may be variable, and as such, sampling on subsequent days is essential prior to excluding infection. In addition, microscopic examination for identification of eggs requires some expertise, and can be time consuming. As such, conduct of population surveys or screening of large groups requires much planning and can be rather cumbersome. Hence, a diagnostic method which is easy to perform as well as being equally or more sensitive and specific than microscopy for the diagnosis of enterobiasis would be most useful for the control of pinworm infection. As such, this study aimed to characterize the antigens of *E. vermicularis* eggs as a preliminary step towards identifying those that would be useful as diagnostic targets for detection in infected individuals.

## Methods

### Sample collection and *E. vermicularis* egg isolation

Anonymized patient samples (perianal swabs) positive for *Enterobius vermicularis* eggs were obtained from the Department of Parasitology, Faculty of Medicine, University of Colombo. A sample of 5 ml blood was provided voluntarily by an anonymous donor diagnosed with chronic enterobiasis (pinworm infection for more than 10 years). All samples had been obtained following informed consent from the participants or their legal guardians. Ethics approval for the study was obtained from the Ethics Review Committee of the Faculty of Medicine, University of Colombo, Sri Lanka (EC-19-034), as per the guidelines published by the Forum of Ethics Review Committees of Sri Lanka^8^, and conducted in accordance between October 2019 and February 2020. Reporting in this manuscript follows the recommendations of the ARRIVE guidelines.

The perianal swabs were made of 2 layers of cellophane and the bottom layer comprised a central, circular, adhesive, clearly marked, blue colour area for easy placement on the perianal skin. *E. vermicularis* eggs attached to these swabs were harvested by using absolute acetone, which was found to be the most effective in detaching eggs from these adhesive tapes. Five different solvents were tested initially: one fourth of the original size of the swab was cut and placed in tubes containing 1 ml of distilled water, absolute alcohol, absolute acetone, phosphate-buffered saline (PBS, pH 7.2) and potassium dichromate (K_2_Cr_2_O_7_), and kept for 5 min at room temperature (RT), while continuously stirring with a pair of forceps. Pre- and post-egg counts were determined by examining each piece of swab under the microscope, and absolute acetone was observed to have successfully removed all eggs attached to the swab.

Extraction of eggs for the study was done thereafter by adding a cut piece of swab, one fourth of the original size of the circular central blue colour area, into 300 µl of acetone in an Eppendorf tube and kept for 1 min at RT whilst stirring with forceps. The swab was examined microscopically under × 100 magnification for any remaining eggs and was re-dipped and stirred until all eggs had been removed into the solution.

### Purification of isolated *E. vermicularis* eggs

The eggs were purified as described below by centrifugation to remove debris, glue and acetone, followed by several steps of washing, in order to prevent any contamination/interference with antigen preparation and the production of immune sera in rats.

Several methods were tested initially to identify the most effective force for pelleting of eggs: using short spins in a mini centrifuge machine with higher centrifugal forces such as 14,000×*g* resulted in an egg clump which could not be reconstituted due to the sticky nature of the eggs and the presence of debris. Therefore, the eluted egg suspension was given increasing forces between 500 and 800×*g* (500×*g*, 650×*g*, 700×*g* and 800×*g*) with 800×*g* able to sediment the eggs effectively. All remaining traces of glue were removed by repetitive washing with 200 µl of acetone and finally with PBS, and then re-suspended in 250 µl of PBS.

### Preparation of *E. vermicularis* egg antigen slides (using intact eggs)

Five microlitres of the re-suspended egg pellet were transferred onto a glass slide and the egg count was estimated (approximately 1000 eggs/250 µl). Final volume of the egg suspension was then adjusted to get approximately 500 eggs per 250 µl. Antigen slides were prepared with 5 μl of egg suspension per spot, containing 5–10 eggs (concentration of 1–2 eggs/μl), placed in the clean wells of a 12-well multi-spot microscope slide. Slides were air-dried overnight at RT. The eggs in each well of the slides were counted and slides were stored at − 70 °C until used.

### Preparation of *E. vermicularis* egg antigen sample (with disrupted/crushed eggs)

Egg antigens were obtained by disrupting the egg shell and release of the larval stage within. Several egg disruption methods were tested using samples of the purified *E. vermicularis* egg suspension to identify the most effective method. Sonicating the egg suspension at 53 kHz with 4 mm glass beads (Marienfeld, Germany) for 30 min at RT and then vortexing for 15–30 min was successful in disrupting all eggs in the test sample.

Vortexing of the egg suspension (250 µl containing approximately 1000 eggs) for 30 min, 45 min or 1 h at repeated intervals, as well as, sonicating of the egg suspension at 53 kHz for 5, 15 or 30 min time periods at RT, resulted in intact eggs in each of the test samples. Mixing the egg suspension either with 1% Triton X-100 in PBS (v/v = 1:1) or with 2% sodium dodecyl sulfate (SDS) in PBS (v/v = 1:1) for 1 min and then vortexing for 5–15 min yielded intact eggs in the former and only partial discharge of larvae in the latter. Three repeated freeze–thaw cycles (15 min at − 80 °C followed by 10 min at 37 °C) of the egg suspension also gave intact eggs, while vortexing with glass beads for 30 min too was unsuccessful. Sonicating at 53 kHz with glass beads for 30 min at RT followed by vortexing for a similar time period finally yielded a considerable proportion of crushed eggs.

### Estimation of the protein concentration of *E. vermicularis* egg antigen sample

**Figure 1:**
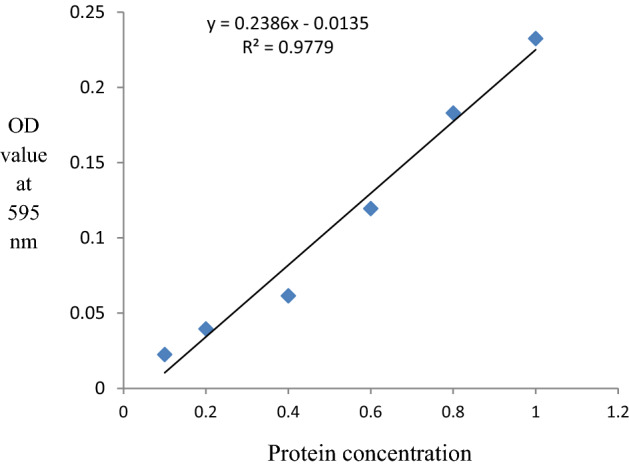
Standard curve of the Bradford assay.

Once the eggs were broken and the antigen prepared, the total protein concentration of the sample was measured using the Bradford assay^9^; with the use of a protein standard and by plotting a calibration curve. Bovine serum albumin (BSA) was used as the protein standard, as it interfered less with the reagents used in the Bradford assay. The extinction coefficient of a dye-albumin complex solution is constant over a tenfold concentration range. Thus, Beer’s law^10^ could be applied for accurate quantification of protein by selecting an appropriate ratio of dye volume to sample concentration. The protein concentration of the test sample was determined by comparison to that of a series of protein standard (BSA) known to reproducibly exhibit a linear absorbance profile in this assay. The optical density (OD) at 595 nm wave-length was measured using a microplate reader and the calibration curve was plotted using a dilution series of BSA standard starting from 10 to 1000 μg (Fig. 1). Then using the standard curve, the protein concentration of the sample was estimated.

### Development of immune serum against *E. vermicularis* egg antigens

Immune serum against *E. vermicularis* egg antigens was developed in a healthy young female adult Wistar rat (approximately 5–6 months old) at the rat facility of the Institute of Biochemistry, Molecular Biology and Biotechnology (IBMBB) of the University of Colombo, where it was housed in a clean polypropylene cage under the following environmental conditions: 22–23 °C temperature, 70% humidity, 12-h light and dark cycles, and with central air conditioning. Feeding was done once in two days with commercially available pelleted (rat) food and clean water (replenished daily) provided through a bottle fixed to the cage. The rat was acclimatized for a week prior to use in the study. Crude egg antigen samples, Freund’s complete (FCA) and Freund’s incomplete adjuvant (FIA) mixtures were made in a hood under sterile conditions. Firstly, 150 μl of egg antigen was transferred into an Eppendorf tube, to which adjuvant was added according to the immunization chart shown in Table 1, to form a total volume of 300 µl. They were mixed more than 30 cycles by sucking in and out using a 1 ml syringe with a 23 G needle and emulsified. A drop from the emulsion was put in water and observed for dispersion in order to ensure it had mixed well.

**Table 1:** Immunization chart.

Day	Volume of antigen (µl)	Volume of FCA (µl)	Volume of FIA (µl)	Total volume (µl)
D0	150	75	75	300
D7	150	30	120	300
D14	150	–	150	300
D28	150	–	150	300

The rat was mildly anaesthetized by placing in a closed desiccator containing anaesthetic ether for a few seconds and then injected with 200–250 μl of antigen-adjuvant mixture subcutaneously using a 1 ml syringe with a 23 G needle as described by Leenaars and Hendriksen^11^. The rat was placed in the cage after regaining consciousness and standard animal care was maintained throughout the process. Weekly booster doses, prepared as indicated in Table 1, were administered on D7, 14 and 28.

Blood was drawn from the immunized rat to obtain serum on Day 0 (pre bleeding), 7, 14, 21, 28, 35 and 42. On each occasion, approximately 450 μl of blood was drawn by tail snipping of the anaesthetized rat. The collected blood was allowed to clot for 1 h at RT and thereafter at 4 °C for 30 min after disturbing the clot. The clotted blood sample was then spun at 950×*g* for 10 min and serum was collected into sterile Eppendorf tubes and stored at − 20 °C until further use.

Each serum sample was de-complemented prior to use for surface immunofluorescence assay, by keeping for 30 min at 56 °C water bath. The de-complemented sera were kept stored at − 20 °C until used.

### Surface immunofluorescence assay

Immunofluorescence assay was performed as described below following the method detailed by Camargo^12^, using the pre-prepared egg antigen slides mentioned above. Rat anti-serum against *E. vermicularis* egg antigens or serum from an individual with chronic enterobiasis were used as the primary antibody, which was then detected by using commercially available fluorescein isothiocyanate (FITC) conjugated goat anti-rat IgG antibodies as the secondary antibody and the slides were examined under a fluorescence microscope.

The egg antigen slides were thawed under dehydrated conditions and each well was incubated with 1:4 dilution of human and rat immune sera in PBS for 45 min at RT. Wells were washed three times with PBS, and thereafter, incubated with 1:40 dilution of the secondary antibody in PBS (goat anti-rat FITC conjugated IgG antibody) for 30 min in the dark at RT. Wells were re-washed with PBS three times in the dark, and then covered with a coverglass and examined under the fluorescence microscope.

### Sodium dodecyl sulfate polyacrylamide gel electrophoresis (SDS-PAGE)

The SDS–PAGE was performed according to the procedure described by Laemmli^13^, and consisted two layers of gel; a resolving (separating) gel layer at the bottom and a stacking gel layer at the top. About 5 ml of 12% acrylamide was used for the separating gel and 2 ml of 5% acrylamide for the stacking gel.

Samples were prepared as follows. Ten microlitres of egg protein at a concentration of 400 μg/ml was mixed with the gel loading buffer at 1:1 ratio and incubated for 5 min at 95 °C. Up to 150 μl of the sample was loaded per well followed by 10 μl of un-stained marker. The gel was run with 1× Tris–Glycine electrophoresis buffer at 70 V for approximately 150 min until the dye front reached the bottom of the gel. A portion of gel containing the protein bands (two lanes with protein marker and a single lane with egg antigens) was removed and silver stained. Remaining portion of gel containing a broad lane of egg antigens was used for the western blot.

Silver staining was done as follows. The gel was firstly incubated in fixing solution containing ethanol, glacial acetic acid and distilled water at a ratio of 30:10:60 respectively for 1 h with gentle shaking. After 30 min, the fixing solution was removed and the gel was incubated in washing solution of 50% methanol in water. The washing solution was then discarded and the gel was incubated in distilled water (rinsing solution), twice for 10 min on each occasion. After removing the rinsing solution, a sensitizing solution of 0.02% sodium thiosulfate in water was added and incubated for 1 min. Then the solution was discarded and the gel was rinsed with distilled water twice, lasting for 1 min each time. After removal of the rinsing solution, the gel was incubated with the freshly prepared staining solution of 0.1% silver nitrate in water for 30 min. After incubation, the gel was washed with de-ionized water and again incubated in freshly prepared developing solution of 2.5% sodium carbonate and 0.02% formaldehyde in water. The reaction was stopped by washing the gel in 1% acetic acid followed by de-ionized water.

### Molecular weight estimation of proteins in SDS-PAGE gel

**Figure 2:**
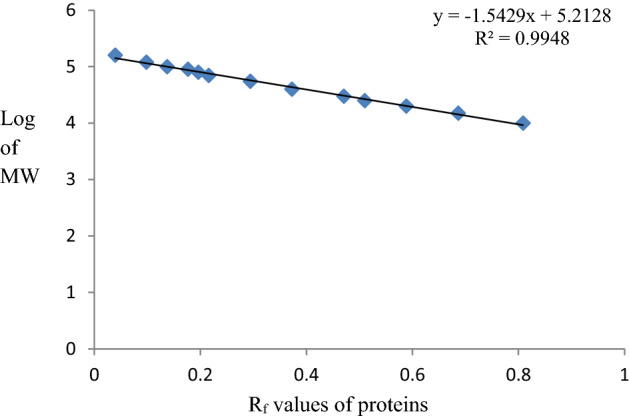
Calibration curve of the molecular weight markers.

Molecular weight estimation was done by plotting the ratio of the migrated distance (in cm) of protein standard of the pre-stained molecular marker to the dye front (R_f_ value) and the log values of molecular weights of the protein standards. BenchMark (Thermo Fisher Scientific, USA) unstained protein ladder was used as the standard molecular weight marker and the dye front of 4.7 cm from the silver staining procedure were used for calculation of R_f_ values (Table 2). The graph plotted using the calculated R_f_ values and log molecular weights is shown in Fig. 2.

**Table 2:** Calculated R_f_ values of the molecular weight (MW) markers.

Band no.	Molecular weight of the marker (Da)	Log values of the MW marker	Migration distance (cm)	R_f_ value
1	10,000	4.0000	3.8	0.8085
2	15,000	4.1761	3.5	0.6863
3	20,000	4.3010	3	0.5882
4	25,000	4.3979	2.6	0.5098
5	30,000	4.4771	2.4	0.4706
6	40,000	4.6021	1.9	0.3725
7	55,000	4.7404	1.5	0.2941
8	70,000	4.8451	1.1	0.2157
9	80,000	4.9031	1	0.1961
10	90,000	4.9542	0.9	0.1765
11	100,000	5.0000	0.7	0.1373
12	120,000	5.0792	0.5	0.0980
13	160,000	5.2041	0.2	0.0392

Molecular weights of the *E. vermicularis* egg protein antigens were then determined by calculating the R_f_ values of each band and comparing them with the calibrated molecular weight markers.

### Western blot analysis

Detection of specific immunogenic proteins in the egg antigens was done using western blotting^14^. The gel containing waffle was placed accurately in the electrode assembly, then closed tightly in the transfer tank with an ice pack, and connected to a power supply. The proteins in the gel were transferred to the nitrocellulose membrane for 2½ h at 70 V.

### Detection of target proteins immobilized on the nitrocellulose membrane

The nitrocellulose membrane was carefully separated into 16 similar pieces and incubated overnight at 4 °C with human and rat sera, each diluted in the ratio of 1:25 with blocking buffer. Thereafter, secondary antibodies conjugated with horse radish peroxidase (HRP) were used. Pre-tested substrate solutions A and B (Solution A: 6 mg 4-Chloro-1-naphthol mixed with 2 ml absolute methanol; Solution B: 6 µl of ice-cold H_2_O_2_ in 10 ml of 0.13 M 1× PBS) that produce a purple colour precipitate on contact with HRP were added to the washed membranes and gently agitated in a platform shaker for 20 min at RT until colour development was visible. Further reaction was stopped by adding water, and the distances of the bands were measured in each membrane.

## Results

### Protein concentration of the *E. vermicularis* egg antigen sample

Concentration of the egg sample was 8000 eggs/ml with an average OD_595nm_ of 0.0805, and the total protein concentration of the crushed egg antigen sample was estimated to be 394 µg/ml.

### Surface immunofluorescence

**Figure 3:**
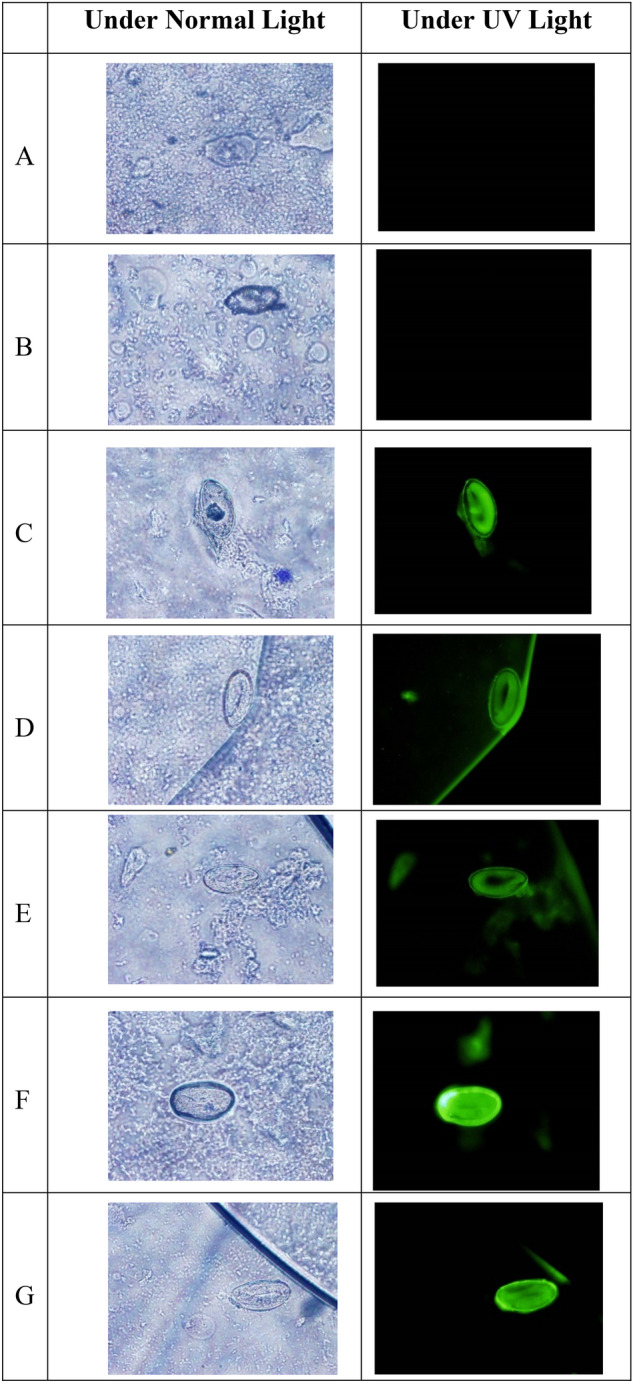
Immunofluorescence of intact eggs with rat serum on (**A**) Day 0, (**B**) Day 7, (**C**) Day 14, (**D**) Day 21, (**E**) Day28, (**F**) Day 35 and (**G**) positive human serum.

Surface immunofluorescence was observed only with rat sera obtained from day 14 till day 42 post-inoculation, indicating that sufficient IgG had formed only by day 14 post-inoculation (Fig. 3A–F). The sample of human serum too gave positive results (Fig. 3G).

### Molecular weights of the *E. vermicularis* egg antigens

**Figure 4:**
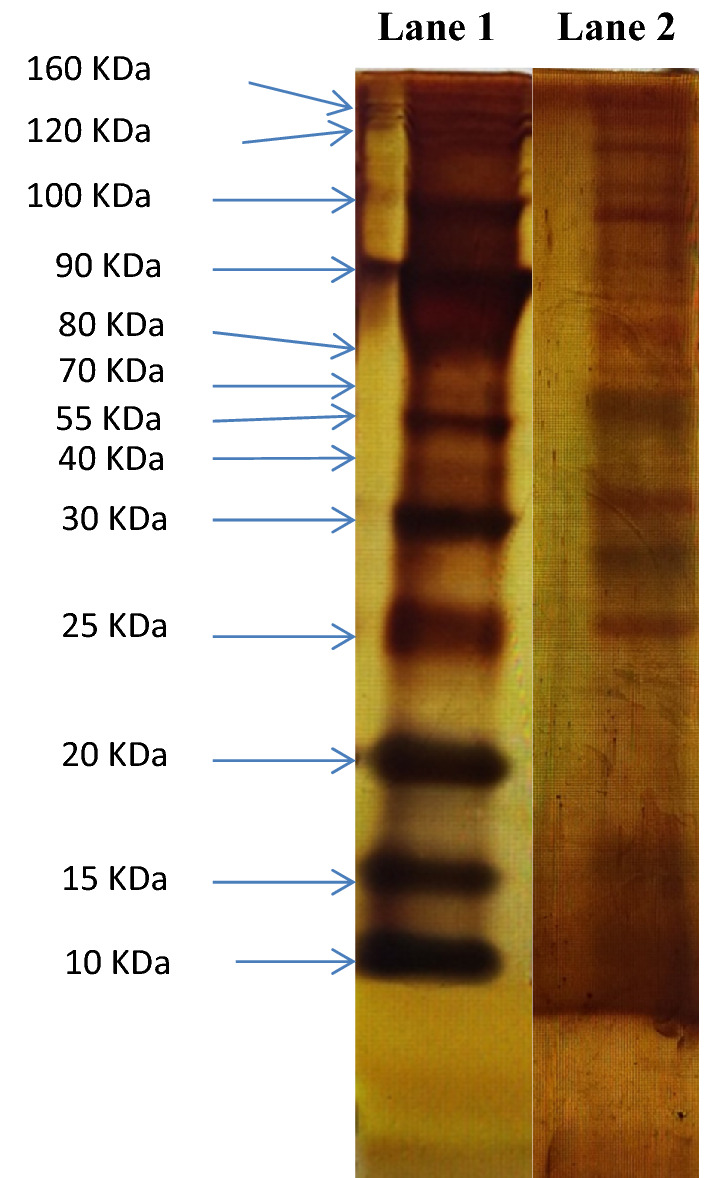
*E. vermicularis* egg antigen bands in silver-stained gel after SDS-PAGE. Lane 1—BenchMark^®^ unstained protein marker (10 kDa to 220 kDa); Lane 2—bands of *E. vermicularis* egg proteins (ranging from 18 to 151 kDa).

About 20 protein bands were detected with approximate molecular weights ranging from 18 to 151 kDa (Fig. 4, Supplementary File S1).

### Western blot analysis

**Figure 5:**
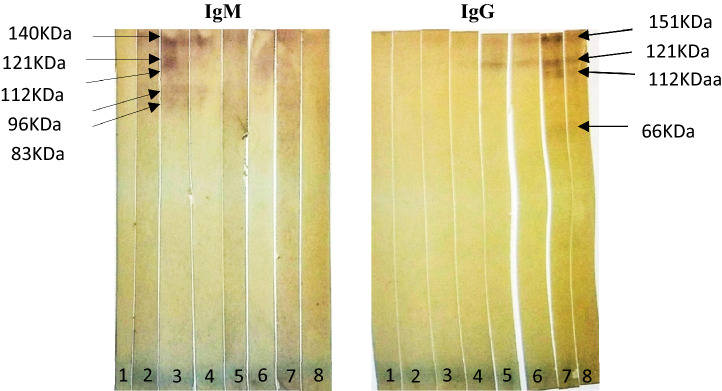
Western blots for IgM and IgG secondary antibodies with, (1) positive human serum, (2) day 0 or negative rat serum, (3) day 7 rat serum, (4) day 14 rat serum, (5) day 21 rat serum, (6) day 28 rat serum, (7) day 35 rat serum, (8) day 42 rat serum.

The secondary antibody dilutions from each incubated strip turned purple on addition of the substrate and the coloured bands that became visible are shown in Fig. 5. Images were edited for brightness, contrast and colour using picture format tools of MS Word to obtain better clarity (un-edited images are shown in Supplementary File S1).

The distances travelled by these blotted bands on the filter papers and their estimated relative molecular weights are given in Table 3. These bands which blotted on the membrane are representative of the immunogenic egg antigens of *E. vermicularis* and would no doubt be useful in future studies directed towards the development of immunodiagnostics for enterobiasis.

**Table 3:** Distances traveled and the estimated molecular weights of the immunogenic *E. vermicularis* egg antigens.

	Positive human serum	Rat serum						
		Day 0	Day 7	Day 14	Day 21	Day 28	Day 35	Day 42
1	–	–	–	–	**0.1 cm—151 kDa**	**0.1 cm—151 kDa**	**0.1 cm—151 kDa**	**0.1 cm—151 kDa**
2	–	–	*0.2 cm—140 kDa*	*0.2 cm—140 kDa*	*0.2 cm—140 kDa*	–	–	–
3	–	–	*0.4 cm—121 kDa*	**0.4 cm***—***121 kDa**	**0.4 cm***—***121 kDa**	**0.4 cm***—***121 kDa**	**0.4 cm***—***121 kDa**	**0.4 cm***—***121 kDa**
4	–	–	*0.5 cm—112 kDa*	–	–	–	**0.5 cm***—***112 kDa**	–
5	–	–	*0.7 cm—96 kDa*	*0.7 cm—96 kDa*	*0.7 cm—96 kDa*	–	–	–
6	–	–	*0.9 cm—83 kDa*	–	*0.9 cm—83 kDa*	–	–	–
7	–	–	–	–	–	–	**1.2 cm***—***66 kDa**	–
8	–	–	–	–	–	–	**2.7 cm***—***21 kDa**	**2.7 cm***—***21 kDa**

IgM: italics, IgG: bold.

## Discussion

Microscopic examination of perianal swabs is the standard method used for diagnosis of enterobiasis^15^. However, the variable and nocturnal nature of egg deposition on the perianal skin by the gravid female *E. vermicularis* worm requires samples to be acquired early morning prior to washing of the perianal region on at least three consecutive days for accurate diagnosis^16,17^. In addition, the swabs need to be examined microscopically by an experienced individual for identification of pinworm eggs, which is rather time consuming. An immunodiagnostic method which is easy to perform with equal or more sensitivity and specificity to microscopy, would be most useful in the treatment and control of this infection. The current study was conducted to identify potential antigens of *E. vermicularis* as a preliminary step towards the development of such an immunodiagnostic tool.

Clinical manifestations of enterobiasis are generally quite suggestive of infection and routine diagnostic tools are fairly reliable^15^. However, many infections may be asymptomatic, with ongoing underlying transmission occurring at individual as well as community level. As healthcare becomes more consumer-focused, the need for convenient and effective point-of-care diagnostics is expanding worldwide. A point-of-care diagnostic tool for enterobiasis could allow for screening and initiation of treatment during a single encounter. In Sri Lanka, mass deworming of schoolchildren conducted through annual school medical inspections for over five decades has been successful in controlling soil-transmitted helminth infections (< 1%) in the country^18^. However, the prevalence of enterobiasis continues to remain over 20% amongst these schoolchildren^3^. As such, a point-of-care diagnostic tool would improve the ease in conduct of epidemiological surveys required to inform public health policy and for implementation of population targeted interventions.

Conduct of the current study for identification of immunogenic surface antigens of *E. vermicularis* eggs was quite challenging due to the lack of scientific literature in this regard. Commencing from the extraction of eggs from perianal swabs, to purification, disruption and then finally the detection of immunogenic antigens required experimentation with many processes and documentation of them in this paper would no doubt be helpful for future research activities in this area.

Scratching off eggs manually from the adhesive cellophane swab was not successful, while absolute alcohol for egg removal was inefficient and caused whitish discolouration of eggs. Absolute acetone (propan-2-one) which is the simplest and smallest ketone, was the best at harvesting these eggs. Acetone exhibits only slight toxicity and was therefore more suitable than other solvents for harvesting pinworm eggs from these adhesive cellophane swabs. Furthermore, purification by serial washing with acetone was also useful in removing any remaining debris and glue flakes from the isolated eggs.

Disruption of the hard and thick egg shell required experimenting with various egg disruptive methods prior to achieving success. Mini spin centrifugation at high centrifugal force for short time periods only resulted in clumping of eggs, while just vortexing or sonicating of eggs was not effective either. Similarly, Triton X-100, which is a widely used detergent to lyse cells for extraction of proteins and organelles and to permeabilize living cell membranes^19^ nor SDS, which is an anionic detergent with a negative charge that usually can denature native proteins by disturbing of non-covalent forces, could disrupt the egg shells successfully. With SDS, it was noted that larval stages did emerge partly from the eggs, but the toxic nature of SDS was thought likely harmful for the rat and to interfere with development of immune sera. Continuous freeze thaw cycles known to damage cells^20^ could not significantly disrupt these pinworm eggs. Finally, the eggs were effectively crushed only with the use of glass beads, together with sonication prior to vortexing helpful in saving time.

Surface immunofluorescence of intact pinworm eggs was clearly observed after reacting with immune serum developed in the Wistar rat as described by Wakayama et al.^21^. Reactivity was observed with sera obtained at day 14 post-inoculation and thereafter, indicative of the presence of immunogenic antigen expression on the surface of pinworm eggs, despite being covered in a hard shell. The positive fluorescence that emanated from human serum of the donor with chronic enterobiasis confirmed that these surface antigens are capable of producing an immune response upon exposure to the host immune system. Surprisingly, this same sample of human serum did not react with the protein bands in the immunoblotting probably due to its greater dilution in this assay (1:25) when compared to that used in the immunofluorescence assay (1:4).

The life cycle of *E. vermicularis* in humans is usually confined to the gut, with onset of infection resulting from ingestion of infective eggs that are deposited on the perianal skin by the gravid female worm^4^. In chronic pinworm infection, repeated exposure to intact eggs, larval stages and adult worms would have occurred in the gut. For the development of immune sera in the rat, crushed eggs were injected subcutaneously, which was successful in yielding an immune response against both intact (immunofluorescence assay) as well as crushed (immunoblotting assay) egg antigens. It thus appears likely that these immunogenic antigens are shared between the different stages of *E. vermicularis*. Furthermore, our results confirm the presence of serum antibodies in persons with chronic enterobiasis.

A total of 20 protein bands of *E. vermicularis* eggs were identified and their approximate molecular weights determined by using SDS-PAGE assay, while their immunogenicity was assessed through immunoblotting with rat antiserum. The most prominent and immunogenic protein bands identified in this study comprised of 8 proteins with molecular weights that ranged between 21 and 151 kDa. The development pattern of IgM and IgG antibodies in rat serum were identified through the immunoblot (Table 3) and conformed to established timelines. IgM antibodies are known to develop within the first two weeks, with peak levels usually on day 7^11^. In our study, IgM against egg antigens were observed on day 7, and remained until day 14–21 in some; while IgG was mainly observed from day 21 onwards and continued to be present until the last day of observation (day 42). If these results can be extrapolated to humans, the presence of specific IgM may be exploited to detect current infection, while IgG would be indicative of exposure to pinworm infection. Autoinfection is common with *E. vermicularis*, and infection is generally known to last for long periods once established due to continued re-infection^22^, thus allowing for the detection of specific IgG. However, the demonstration of more than twofold rise in titres of specific IgG may be required to differentiate between current and past infection.

The possibility of detecting these immunogenic antigens in blood or other human tissues needs further study. In-depth analysis on the presence and detection of these immunogenic antigens in various body tissues, the duration of their availability, as well as specificity (with no cross reactivity to other helminth antigens) may provide an opportunity for the design and development of novel, rapid, point-of-care diagnostics for *E. vermicularis* infection. Furthermore, recent studies have discovered the immune-modulation properties of tissue-invasive helminths and their excretory-secretory molecules, which are now being exploited for use as immune therapeutics^23–26^. Several studies have suggested an immune-protective role of *E. vermicularis* infection against allergies^27–29^. As such, understanding the effects yielded by these *E. vermicularis* antigens on the host immune system (including gut mucosal immunity) may create opportunities for the design of novel therapeutics and vaccines.

In conclusion, the methods used in this study were effective in obtaining *E. vermicularis* egg antigens which were immunogenic. Furthermore, surface antigens of intact eggs reacted with antibodies developed against crushed egg antigens. These findings may pave the way for the development of effective immunodiagnostics.

## Supplementary Information

Supplementary Information 1.

Supplementary Information 2.

## Data Availability

All data generated or analysed during this study are included in this published article [and its Supplementary Information files].
