# Supplementary material for: Characterization of antigens of Enterobius vermicularis (pinworm) eggs
Source: Sci Rep. 2022 Aug 24;12:14414. doi: 10.1038/s41598-022-18303-8 (PMC9402560; doi:10.1038/s41598-022-18303-8)
Supplement: Supplementary file 1 — Supplementary Information 1. [file 41598_2022_18303_MOESM1_ESM.docx]

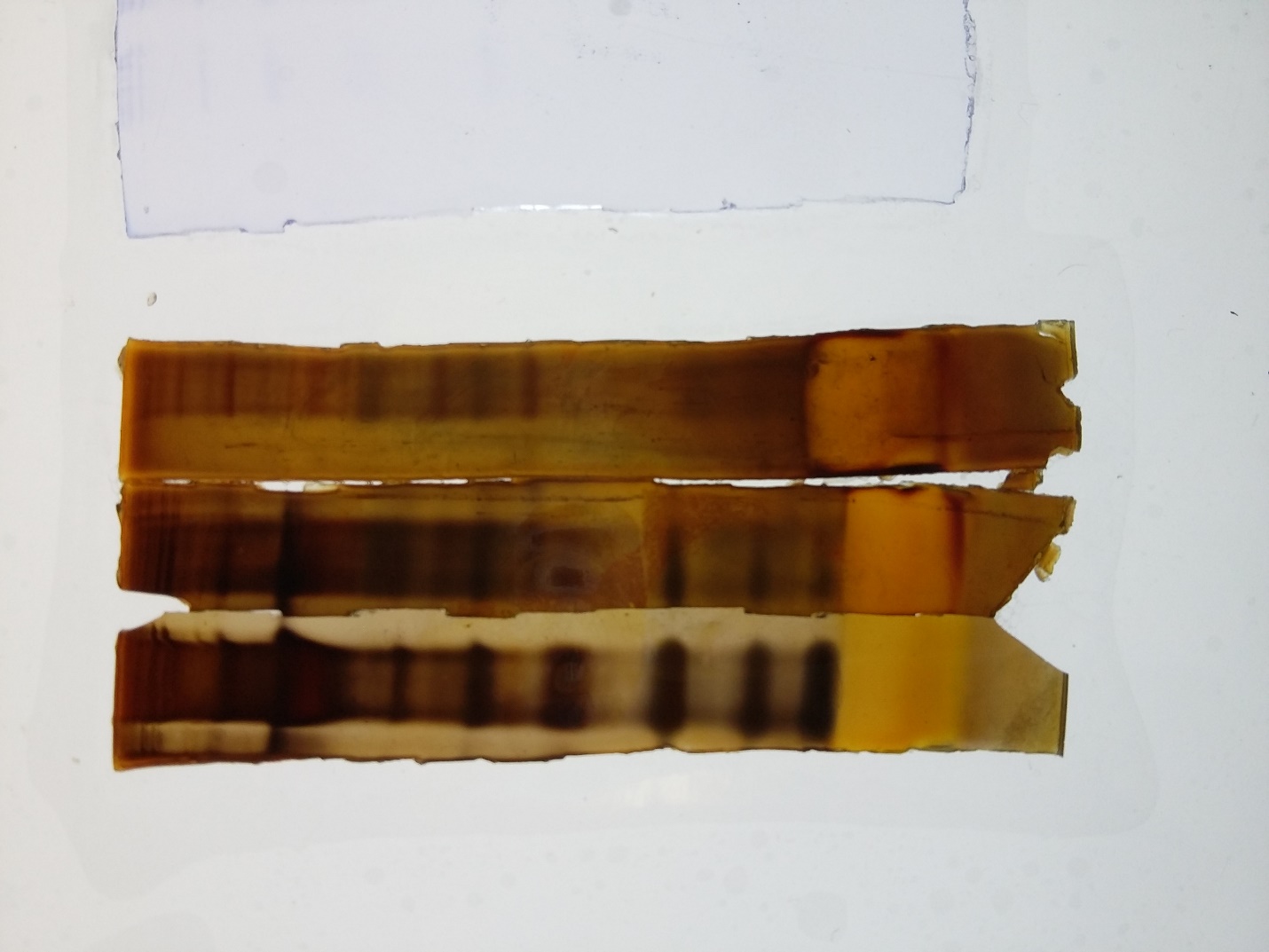


Figure 4a (Un-edited): *E. vermicularis* egg antigen bands in silver-stained gel after SDS-PAGE.

Lanes 1 and 2 - BenchMark® unstained protein marker (10 KDa to 220 KDa);

Lane 3 - bands of *E. vermicularis* egg proteins (ranging from 18 – 151 KDa).


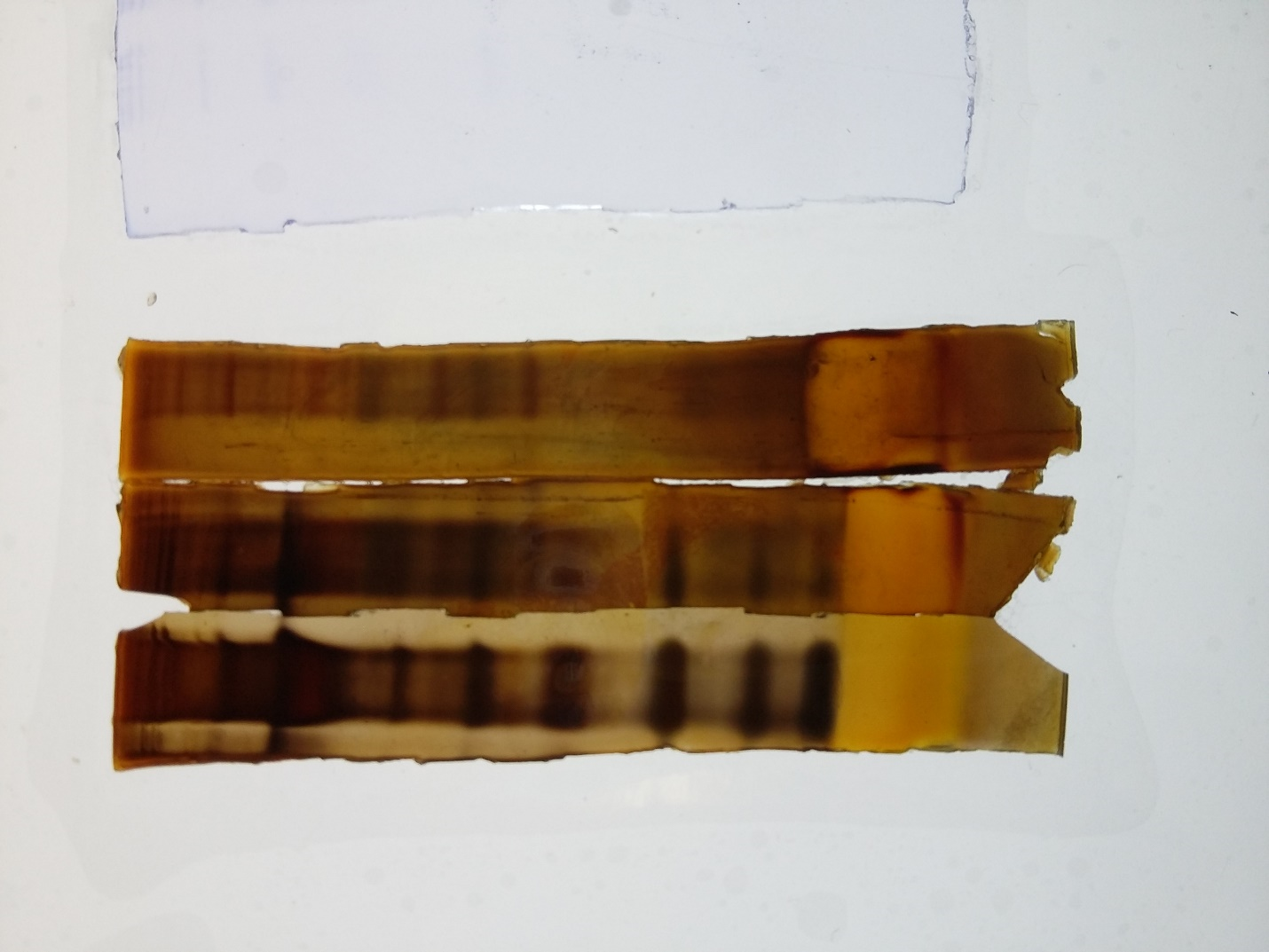


160 KDa

120 KDa

100 KDa

90 KDa

80 KDa

70 KDa

55 KDa

40 KDa

30 KDa

25 KDa

20 KDa

15 KDa

10 KDa

Lane 1

Lane 2

Lane 3

Figure 4b (Edited): *E. vermicularis* egg antigen bands in silver-stained gel after SDS-PAGE.

Lanes 1 and 2 - BenchMark® unstained protein marker (10 KDa to 220 KDa);

Lane 3 - bands of *E. vermicularis* egg proteins (ranging from 18 – 151 KDa).


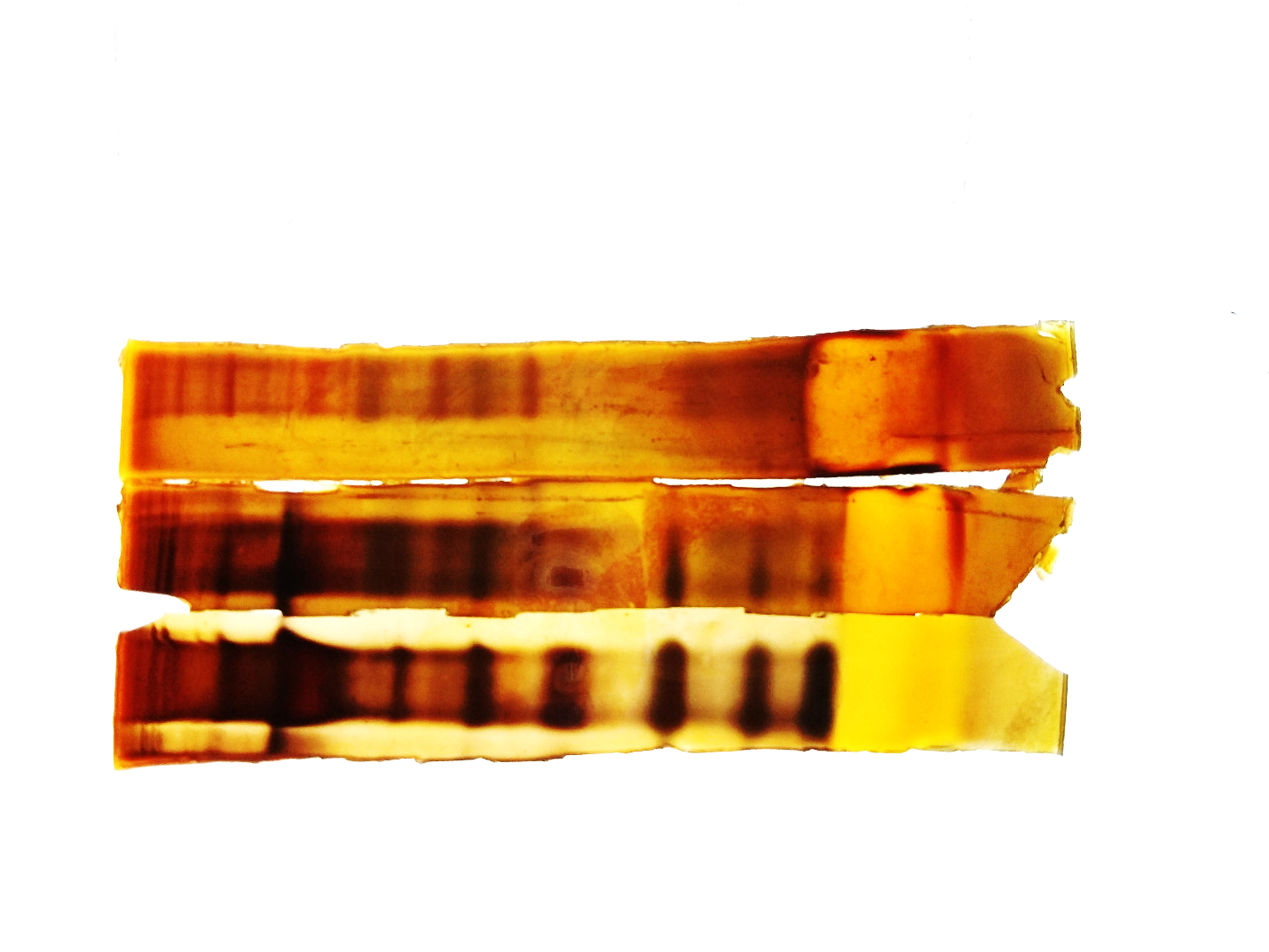


160 KDa

120 KDa

100 KDa

90 KDa

80 KDa

70 KDa

55 KDa

40 KDa

30 KDa

25 KDa

20 KDa

15 KDa

10 KDa

Lane 1

Lane 2

Lane 3

Figure 4c (Enhanced - Brightness +40% & Contrast 40%): *E. vermicularis* egg antigen bands in silver-stained gel after SDS-PAGE.

Brightness +40% & contrast 40%

Lanes 1 and 2 - BenchMark® unstained protein marker (10 KDa to 220 KDa);

Lane 3 - bands of *E. vermicularis* egg proteins (ranging from 18 – 151 KDa).


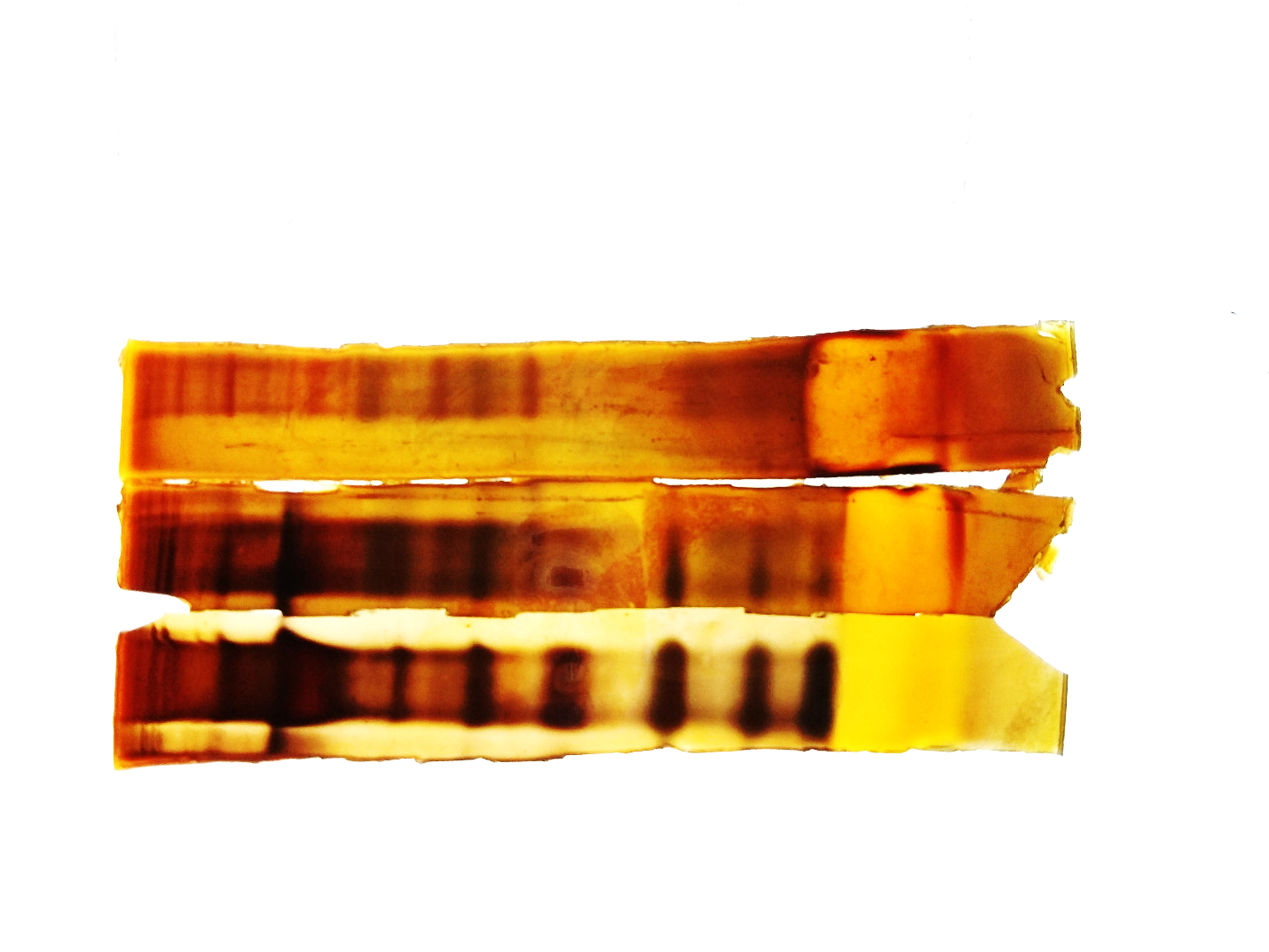


160 KDa

120 KDa

100 KDa

90 KDa

80 KDa

70 KDa

55 KDa

40 KDa

30 KDa

25 KDa

20 KDa

15 KDa

10 KDa

Lane 1

Lane 2

Lane 3

Figure 4d (Enhanced - Brightness +40% & Contrast 40%): *E. vermicularis* egg antigen bands in silver-stained gel after SDS-PAGE.

Lanes 1 and 2 - BenchMark® unstained protein marker (10 KDa to 220 KDa);

Lane 3 - bands of *E. vermicularis* egg proteins (ranging from 18 – 151 KDa).
