# Supplementary material for: Characterization of antigens of Enterobius vermicularis (pinworm) eggs
Source: Sci Rep. 2022 Aug 24;12:14414. doi: 10.1038/s41598-022-18303-8 (PMC9402560; doi:10.1038/s41598-022-18303-8)
Supplement: Supplementary file 2 — Supplementary Information 2. [file 41598_2022_18303_MOESM2_ESM.docx]

Lane 3

Lane 2

Lane 1


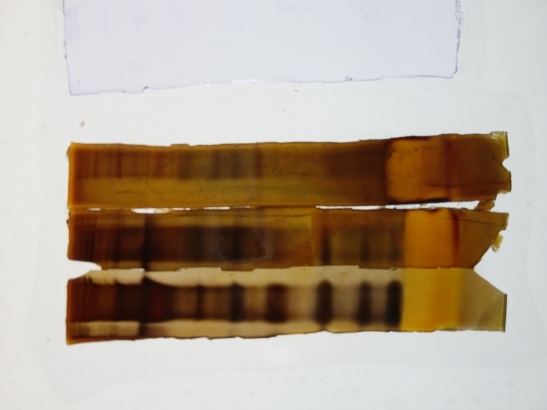


Figure 4 (Un-edited): *E. vermicularis* egg antigen bands in silver-stained gel after SDS-PAGE.

Lanes 1 and 2 - BenchMark® unstained protein marker (10 KDa to 220 KDa);

Lane 3 - bands of *E. vermicularis* egg proteins (ranging from 18 – 151 KDa).

**Only Lanes 1 and 3 are shown in the manuscript.**


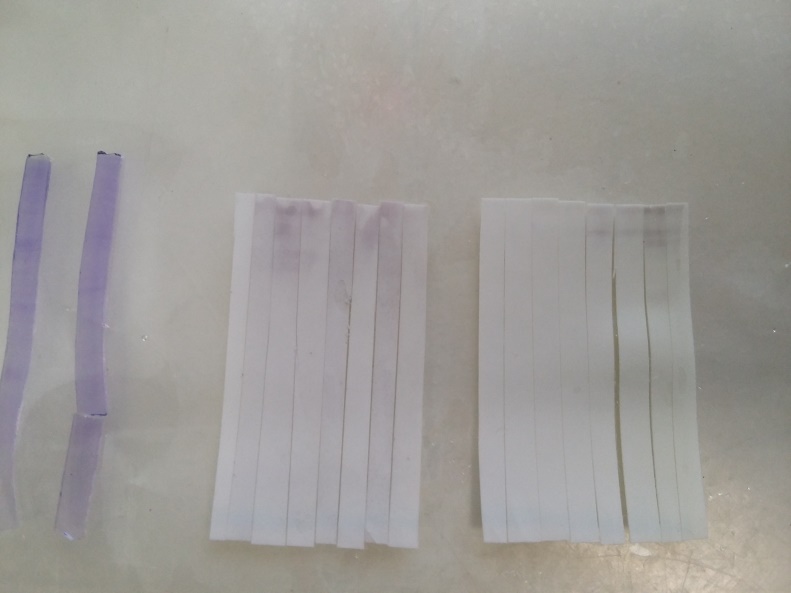


2

8

7

6

5

4

3

1

8

7

6

3

5

4

2

1

**IgG**

**IgM**

Figure 5 (Un-edited): Western blots for IgM and IgG secondary antibodies with, (1) positive human serum, (2) day 0 or negative rat serum, (3) day 7 rat serum, (4) day 14 rat serum, (5) day 21 rat serum, (6) day 28 rat serum, (7) day 35 rat serum, (8) day 42 rat serum (nitrocellulose membrane was cut into 16 strips prior to hybridisation with primary and secondary antibodies).
